# Proposal of a Taxonomic Nomenclature for the Bacillus cereus Group Which Reconciles Genomic Definitions of Bacterial Species with Clinical and Industrial Phenotypes

**DOI:** 10.1128/mBio.00034-20

**Published:** 2020-02-25

**Authors:** Laura M. Carroll, Martin Wiedmann, Jasna Kovac

**Affiliations:** a Structural and Computational Biology Unit, EMBL, Heidelberg, Germany; b Department of Food Science, Cornell University, Ithaca, New York, USA; c Department of Food Science, The Pennsylvania State University, University Park, Pennsylvania, USA; University of Queensland

**Keywords:** *Bacillus anthracis*, *Bacillus cereus*, *Bacillus cereus* group, *Bacillus thuringiensis*, bioterrorism, foodborne pathogens, phylogenetic analysis, taxonomy

## Abstract

The Bacillus cereus group comprises numerous closely related species, including bioterrorism agent B. anthracis, foodborne pathogen B. cereus, and biopesticide B. thuringiensis. Differentiating organisms capable of causing illness or death from those used in industry is essential for risk assessment and outbreak preparedness. However, current species definitions facilitate species-phenotype incongruences, particularly when horizontally acquired genes are responsible for a phenotype. Using all publicly available B. cereus group genomes (*n *= 2,231), we show that current species definitions lead to overlapping genomospecies clusters, in which 66.2% of genomes belong to multiple genomospecies at a conventional 95 average nucleotide identity (ANI) genomospecies threshold. A genomospecies threshold of ≈92.5 ANI is shown to reflect a natural gap in genome similarity for the B. cereus group, and medoid genomes identified at this threshold are shown to yield resolvable genomospecies clusters with minimal overlap (six of 2,231 genomes assigned to multiple genomospecies; 0.269%). We thus propose a nomenclatural framework for the B. cereus group which accounts for (i) genomospecies using resolvable genomospecies clusters obtained at ≈92.5 ANI, (ii) established lineages of medical importance using a formal collection of subspecies names, and (iii) heterogeneity of clinically and industrially important phenotypes using a formalized and extended collection of biovar terms. We anticipate that the proposed nomenclature will remain interpretable to clinicians, without sacrificing genomic species definitions, which can in turn aid in pathogen surveillance; early detection of emerging, high-risk genotypes; and outbreak preparedness.

## Abstract (precis)

Historical species definitions for many prokaryotes, including pathogens, have relied on phenotypic characteristics that are inconsistent with genome evolution. This scenario forces microbiologists and clinicians to face a tradeoff between taxonomic rigor and clinical interpretability. Using the Bacillus cereus group as a model, a conceptual framework for the taxonomic delineation of prokaryotes which reconciles genomic definitions of species with clinically and industrially relevant phenotypes is presented. The nomenclatural framework outlined here serves as a model for genomics-based bacterial taxonomy that moves beyond arbitrarily set genomospecies thresholds while maintaining congruence with phenotypes and historically important species names.

## INTRODUCTION

Historically, prokaryotic species have been defined using various methods (e.g., phenotypic characterization, 16S rRNA gene sequencing, and DNA-DNA hybridization) (1–3). However, contemporary species delineation practices have migrated to high-throughput, *in silico* average nucleotide identity (ANI)-based methods (4), for which two genomes belong to the same genomospecies if they share an ANI value above a set threshold (usually 95 ANI) (5). Paradoxically, evolutionary insights provided by ANI-based species delineation can lead to greater taxonomic ambiguity, as species names deeply ingrained in medicine and industry may be inconsistent with genome evolution (6–8). In these cases, microbiologists face a tradeoff: revise the taxonomy to reflect genomic differences, potentially sacrificing clinical interpretability, or continue to use established species names and ignore underlying genomic diversity.

The Bacillus cereus group, also known as B. cereus sensu lato, is one such species complex plagued by taxonomic inconsistencies. Notable members include B. anthracis, the etiological agent of anthrax and renowned bioterrorism agent (9–12); B. cereus sensu stricto, which is commonly regarded as a foodborne pathogen but has been associated with anthrax-like symptoms and other severe infections (13, 14); and B. thuringiensis, a popular industrial biopesticide control agent (15, 16). Phenotypic characteristics used for taxonomic assignment of B. cereus group species (e.g., motility and hemolysis) vary within and among species (1, 2, 17, 18). Furthermore, genomic determinants responsible for some phenotypes are plasmid mediated, such as synthesis of anthrax toxin/capsular proteins (19–22), bioinsecticidal crystal proteins (23–25), and emetic toxin (cereulide) synthetase proteins (26, 27). These traits can be lost, gained, heterogeneous in their presence within a species, or present across multiple species (28–31).

ANI-based genomospecies assignment, however, has done little to alleviate taxonomic ambiguity. An influx of novel B. cereus sensu lato species (three published between 2013 and 2016 [32–34] and nine in 2017 [35]) has relied on variable genomospecies thresholds ranging from 92 to 96 ANI (33–35). This can lead to overlapping genomospecies clusters where some genomes may belong to more than one genomospecies, depending on the threshold used. Further confusion arises when “novel” species encompass established lineages within their genomospecies thresholds. For example, *B. paranthracis*, a species published in 2017 (35), encompasses the established foodborne pathogen known as emetic “B. cereus” (13, 36–38) within its genomospecies boundaries at a conventional 95 ANI threshold (39).

Current species definitions do not account for species-phenotype incongruences, which can potentially lead to high-consequence misclassifications of an isolate’s virulence potential. For example, clinical diagnostics used to rule out the presence of B. anthracis (1, 2) may incorrectly exclude an anthrax-causing strain exhibiting phenotypic characteristics associated with “B. cereus” as the cause of illness (30, 40–42). Additionally, the ability to cause anthrax is attenuated in B. anthracis strains which lack genes required for anthrax toxin and capsule formation (43). The problem at hand requires the construction of an ontological framework which is accurate in terms of its adherence to widely accepted genomic and taxonomic definitions of bacterial genomospecies while still being informative, intuitive, and actionable to those in public health and industry. Here, we leverage all publicly available assembled B. cereus group genomes (*n *= 2,231) to construct a phylogenomically informed taxonomic framework with the flexibility to account for phenotypes of interest to those in public health and industry.

## RESULTS

### Current species definitions cannot reliably differentiate B. anthracis from neighboring lineages.

**FIG 1:**
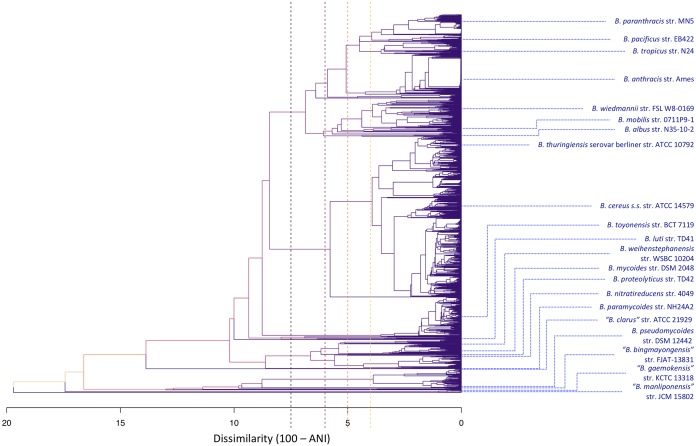
Dendrogram constructed using symmetric pairwise average nucleotide identity (ANI) dissimilarities calculated between 2,218 B. cereus group genomes from NCBI’s RefSeq database with *N*_50_ of >20 kbp (i.e., $D_{\text{ANI}}^{\text{sym}}$ in Materials and Methods) and the average linkage hierarchical clustering method implemented in the hclust function in R. Blue tip labels denote the location of species type strain/reference genomes in the dendrogram, while tree height corresponds to ANI dissimilarity. Branch colors correspond to branch height within the tree. Dashed vertical lines appear at dissimilarities of 7.5, 6, 5, and 4, which correspond to ANI thresholds of 92.5, 94, 95, and 96, respectively (from left to right in order of appearance along the *x* axis).

**FIG 2:**
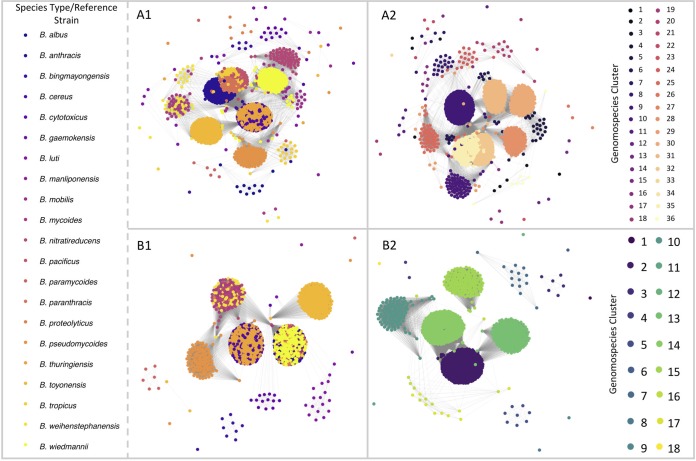
Weighted undirected graphs constructed using symmetric pairwise average nucleotide identity (ANI) values calculated between 2,218 B. cereus group genomes from NCBI’s RefSeq database with *N*_50_ of >20 kbp (i.e., $S_{\text{ANI}}^{\text{sym}}$ in Materials and Methods). Nodes represent individual genomes, while weighted edges connect each pair of genomes with a mean ANI value of ≥95 (A) and ≥92.5 (B), where edge weight corresponds to the mean ANI value of the pair. Nodes (i.e., genomes) are colored by (i) closest matching type strain genome or (ii) closest matching medoid genome of clusters formed at the respective ANI value. Graphs were constructed using the graphout layout algorithm implemented in R’s igraph package, using 1 million iterations and a charge of 0.02.

The practice of calculating ANI values between a genome of interest and the genomes of known B. cereus group species type strains (see Table S1 in the supplemental material) (33–35, 39) and using the widely accepted threshold of 95 ANI (5) as a hard genomospecies cutoff produced nonoverlapping genomospecies clusters for Bacillus albus, “*B. bingmayongensis*,” B. cytotoxicus, “*B. gaemokensis*,” B. luti, “*B. manliponensis*,” B. nitratireducens, B. paramycoides, B. proteolyticus, B. pseudomycoides, and B. toyonensis (Table S2). No genomes assigned to these genomospecies shared ≥95 ANI with any genomes assigned to a different genomospecies (Fig. 1 and 2A1). However, several type strain-centric genomospecies overlapped, including clusters formed by the type strains of (i) B. cereus sensu stricto and B. thuringiensis and (ii) B. mycoides and B. weihenstephanensis, as has been documented previously (Fig. 1 and 2A1; Table S2) (29, 34, 44). The type strains of B. mobilis and B. wiedmannii also produced overlapping genomospecies in which a genome could share ≥95 ANI with both species type strains (Fig. 1 and 2A1; Table S2). The largest source of ambiguity, however, stemmed from B. anthracis and neighboring lineages, as the genomospecies cluster formed by the B. anthracis reference genome overlapped with those of B. pacificus, B. paranthracis, and B. tropicus (Fig. 1 and 2A1; Table S2).

10.1128/mBio.00034-20.3 TABLE S1 Species type strain/NCBI RefSeq reference genomes for 18 currently recognized B. cereus group species and three published putative species used in this study. Download Table S1, XLSX file, 0.01 MB. Copyright © 2020 Carroll et al. 2020 Carroll et al. This content is distributed under the terms of the Creative Commons Attribution 4.0 International license.

10.1128/mBio.00034-20.4 TABLE S2 Proportion of genomes assigned to genomospecies X (rows) using the species type strain/reference genome at a threshold of 95 ANI which share ≥95 ANI with one or more genomes assigned to genomospecies Y (columns). Download Table S2, XLSX file, 0.01 MB. Copyright © 2020 Carroll et al. 2020 Carroll et al. This content is distributed under the terms of the Creative Commons Attribution 4.0 International license.

The species overlap problem persisted at 95 ANI when medoid genomes were used to construct genomospecies clusters (Fig. 2A2; Table S3). All genomospecies which were nonoverlapping when type strains were used (e.g., B. pseudomycoides and *B. toyonensis*) remained nonoverlapping, except for *B. proteolyticus* (Fig. 2A2; Table S3). All overlapping genomospecies continued to produce multispecies classifications at 95 ANI, albeit at a lower rate than type strain-centric clusters: 405 (18.2%) and 1,478 (66.2%) genomes were assigned to 2 or more medoid- or type strain-centric genomospecies, respectively (Fig. 2A).

10.1128/mBio.00034-20.5 TABLE S3 Proportion of genomes assigned to genomospecies X (rows) using the medoid genome at a threshold of 95 ANI which share ≥95 ANI with one or more genomes assigned to genomospecies Y (columns). Download Table S3, XLSX file, 0.02 MB. Copyright © 2020 Carroll et al. 2020 Carroll et al. This content is distributed under the terms of the Creative Commons Attribution 4.0 International license.

### Genomic elements responsible for anthrax, emetic, and insecticidal toxin production exhibit heterogeneous presence in multiple species using current genomospecies definitions.

**FIG 3:**
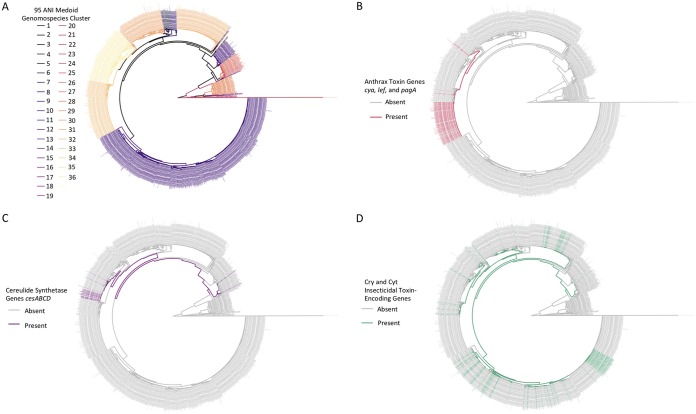
Maximum likelihood phylogenies of 2,218 B. cereus group genomes with *N*_50_ of >20 kbp. Tip and branch labels are colored by genomospecies assignment using medoid genomes of genomospecies clusters formed at the widely used genomospecies threshold of 95 ANI (clusters are arbitrarily numbered) (A) and presence (colored) and absence (gray) of anthrax toxin genes *cya*, *lef*, and *pagA* (B); cereulide synthetase-encoding *cesABCD* (C); and one or more previously described Cry or Cyt insecticidal toxin-encoding genes (D). Phylogenies were constructed using core SNPs identified in 79 single-copy orthologous gene clusters present in 2,231 B. cereus group genomes. The type strain of “*B. manliponensis*” (i.e., the most distantly related member of the group) was treated as an outgroup on which each phylogeny was rooted. Virulence genes (*cya*, *lef*, and *pagA* and *cesABCD*) were detected using BTyper version 2.3.2 (default thresholds), while insecticidal toxin-encoding genes were detected using BtToxin_scanner version 1.0 (default settings; presence and absence of high-confidence, previously known Cry- and Cyt-encoding genes are shown, with predicted putative novel insecticidal toxin-encoding genes excluded).

Additional nomenclatural discrepancies arise when a trait of interest is plasmid encoded, such as anthrax toxin genes *cya* (edema factor encoding), *lef* (lethal factor encoding), and *pagA* (protective antigen encoding) (45): 93 of 241 (38.6%) genomes most closely resembling the B. anthracis reference genome at ≥95 ANI did not possess anthrax toxin genes (Fig. 3A and B; Table S4). Notably, isolates which most closely resemble B. anthracis by current species definitions (i.e., ≥95 ANI), despite lacking anthrax toxin-encoding genes, do not appear to be uncommon. Such strains have been isolated from diverse environments (e.g., soil, animal feed, milk, spices, egg whites, and baby wipes) and from six continents, plus the International Space Station (Table S4). The classification of these isolates as B. anthracis could lead to incorrect assumptions of their anthrax-causing capabilities.

10.1128/mBio.00034-20.6 TABLE S4 Genomes most closely resembling the B. anthracis strain Ames species reference genome with ≥95 ANI in which anthrax toxin genes *cya*, *lef*, and *pagA* were not detected. Download Table S4, XLSX file, 0.02 MB. Copyright © 2020 Carroll et al. 2020 Carroll et al. This content is distributed under the terms of the Creative Commons Attribution 4.0 International license.

Importantly, isolates which display phenotypic characteristics associated with “B. cereus” (e.g., motility and gamma bacteriophage resistance) can cause anthrax (2, 30, 31, 40–42). Despite the assertion that it is a clonal species with low diversity (46–48), the B. anthracis genomospecies cluster formed at 95 ANI encompasses lineages which fall outside the one most commonly associated with anthrax illness (Fig. 3A and B). At a 95 ANI genomospecies threshold, three of seven genomes deposited in RefSeq as anthrax-causing “B. cereus” most closely resembled the B. anthracis reference genome (Fig. 3A and B; Table S5), while also sharing ≥95 ANI with the *B. paranthracis* type strain genome (Table S5). The remaining four anthrax-causing “B. cereus” genomes most closely resembled the *B. tropicus* type strain, shared ≥95 ANI with the *B. paranthracis* type strain, and shared between 94 and 95 ANI with the B. anthracis species reference genome (Table S5). The separation of anthrax-causing “B. cereus” genomes into two genomospecies at 95 ANI was maintained when medoid genomes were used (Fig. 2A2; Table S5). As such, several anthrax-causing “B. cereus” strains are technically still B. anthracis at 95 ANI (Fig. 3A and B) and despite having a mosaic of phenotypic characteristics attributed to “B. cereus” and B. anthracis.

10.1128/mBio.00034-20.7 TABLE S5 Genomes submitted to NCBI’s RefSeq database as anthrax-causing “B. cereus.” Download Table S5, XLSX file, 0.01 MB. Copyright © 2020 Carroll et al. 2020 Carroll et al. This content is distributed under the terms of the Creative Commons Attribution 4.0 International license.

Similar issues plague emetic “B. cereus,” designated as such by its ability to produce cereulide, a toxin responsible for foodborne illness characterized by vomiting symptoms (13, 38, 49). At 95 ANI, all 30 emetic “B. cereus” genomes most closely resembled the *B. paranthracis* type strain, were confined to a single medoid-centric genomospecies, and were interspersed among genomes which lacked cereulide synthetase-encoding genes *cesABCD* (Fig. 3A and C; Table S6). *cesABCD* were detected in five genomes representing two additional medoid-based genomospecies at 95 ANI (Fig. 3A and C; Table S6). One contained the type strains of B. weihenstephanensis and B. mycoides, which is unsurprising considering that cereulide-producing B. weihenstephanensis has been isolated in rare cases (28, 50). However, two genomes categorized previously as emetic “B. weihenstephanensis” belonged to a completely separate genomospecies at 95 ANI (Fig. 3A and C; Table S6).

10.1128/mBio.00034-20.8 TABLE S6 Genomes used in this study (*n *= 2,231). Download Table S6, XLSX file, 0.5 MB. Copyright © 2020 Carroll et al. 2020 Carroll et al. This content is distributed under the terms of the Creative Commons Attribution 4.0 International license.

The Cry and Cyt insecticidal proteins associated with B. thuringiensis (i.e., Bt toxins), which can be plasmid mediated, face similar issues, as B. thuringiensis has historically been defined by its ability to produce insecticidal toxins (e.g., Cry and Cyt toxins) (51). However, genes encoding known insecticidal toxins were detected in nine of 21 B. cereus group type strain-centric genomospecies at 95 ANI (Fig. 3A and D). These results are consistent with previous findings, as Bt toxin production has been attributed to numerous lineages (29, 51, 52).

### ANI-based comparisons to medoid genomes using a lowered genomospecies threshold of ≈92.5 eliminate the species overlap problem.

**FIG 4:**
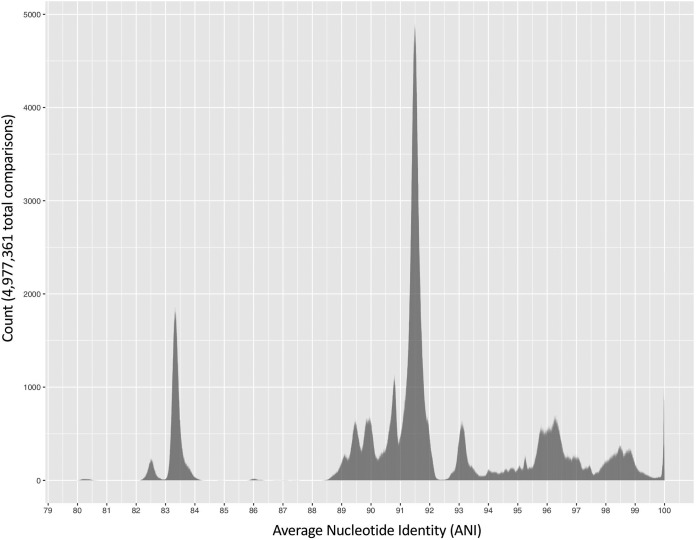
Histogram of pairwise average nucleotide identity (ANI) values calculated between 2,231 B. cereus group genomes downloaded from NCBI’s RefSeq database. FastANI version 1.0 was used to calculate all pairwise ANI values. For histograms colored according to closest species type strain/reference genome at a conventional ≥95 ANI threshold, or histograms showing pairwise ANI values calculated between genomes meeting additional quality thresholds, see Fig. S1 and S2, respectively.

**FIG 5:**
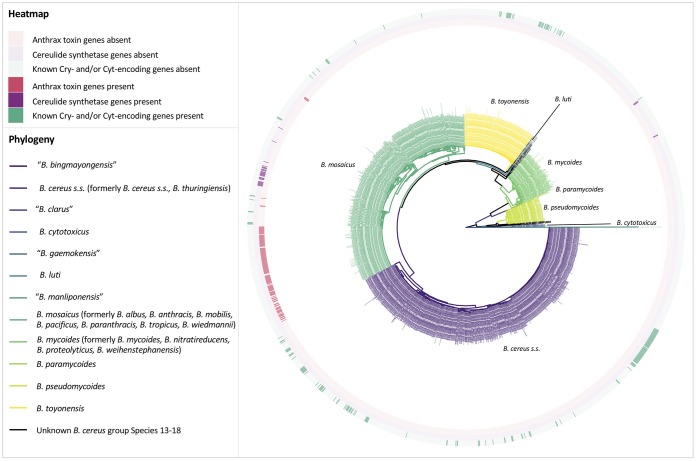
Maximum likelihood phylogeny of 2,218 B. cereus group genomes with *N*_50_ of >20 kb. Tip and branch labels are colored by genomospecies assignment using medoid genomes of genomospecies clusters formed at the proposed genomospecies threshold of 92.5 ANI. Phylogeny was constructed using core SNPs identified in 79 single-copy orthologous gene clusters present in 2,231 B. cereus group genomes. The type strain of “*B. manliponensis*” (i.e., the most distantly related member of the group) was treated as an outgroup on which the phylogeny was rooted.

Numerous bacterial genomospecies have showcased a breakpoint in genome similarity which is close to 95 ANI (5); however, ANI values among a significant proportion of B. cereus group genomes, particularly B. anthracis and neighboring lineages, fall within the 93 to 95 ANI range, with a breakpoint occurring at ≈92.5 ANI (Fig. 4; Fig. S1). Using a hard 92.5 ANI threshold for B. cereus group genomospecies assignment, rather than 95, nearly eliminates the species overlap problem: only six of 2,231 genomes (0.269%) were assigned to 2 or more medoid-based genomospecies (Fig. 2B2; Table S7), compared to 18.2% and 66.2% of genomes assigned to multiple genomospecies at 95 ANI when medoid genomes and species type strain/reference genomes were used, respectively (Fig. 2; Tables S2 and S3). Eighteen genomospecies were present at a 92.5 ANI threshold, compared to 36 medoid-centric genomospecies at 95 ANI (Fig. 3A and Fig. 5; Tables S3 and S7). Notably, at 92.5 ANI, seven genomospecies did not possess type strains of any published species (Table S7), indicating that putative novel genomospecies may be present. While one of these genomospecies has recently been proposed as novel species “B. clarus” (53), the remaining six are uncharacterized (Table S8).

10.1128/mBio.00034-20.1 FIG S1 Histograms of pairwise ANI values between 2,218 B. cereus group genomes with *N*_50_ of >20 kbp. For each of 18 published species and 3 proposed putative species, ANI values calculated between two genomes for which one or more genome(s) shared ≥95 ANI with the species type strain or reference genome are colored in pink. ANI values calculated between two genomes for which both genomes shared <95 ANI with the species type strain/reference genome are colored in gray. See Table S1 for details regarding species type strain genomes. Download FIG S1, PDF file, 0.5 MB. Copyright © 2020 Carroll et al. 2020 Carroll et al. This content is distributed under the terms of the Creative Commons Attribution 4.0 International license.

10.1128/mBio.00034-20.9 TABLE S7 Proportion of genomes assigned to genomospecies X (rows) using the medoid genome at a threshold of 92.5 ANI which share ≥92.5 ANI with one or more genomes assigned to genomospecies Y (columns). Download Table S7, XLSX file, 0.01 MB. Copyright © 2020 Carroll et al. 2020 Carroll et al. This content is distributed under the terms of the Creative Commons Attribution 4.0 International license.

10.1128/mBio.00034-20.10 TABLE S8 Genomes belonging to one of six previously unpublished genomospecies clusters formed at ANI of ≥92.5 (i.e., putative novel genomospecies). Download Table S8, XLSX file, 0.01 MB. Copyright © 2020 Carroll et al. 2020 Carroll et al. This content is distributed under the terms of the Creative Commons Attribution 4.0 International license.

10.1128/mBio.00034-20.2 FIG S2 Sample of histograms constructed using pairwise ANI values calculated between B. cereus group genomes meeting various quality thresholds. Because distribution breakpoints and shape were robust to the exclusion of genomes at all tested thresholds, the six histograms shown represent pairwise ANI values calculated between raw genomes downloaded directly from NCBI’s RefSeq database (i.e., the least stringent quality thresholds, with no filtering criteria employed; *n *= 2,231 genomes) (A), genomes with *N*_50_ of >20 kb (the set of genomes from which medoid genomes were selected; *n *= 2,218) (B), genomes with *N*_50_ of >0 and no contigs assigned to a domain other than *Bacteria* (*n *= 1,291) (C), genomes with *N*_50_ of >20 kb and no contigs assigned to a domain other than *Bacteria* (*n *= 1,289) (D), genomes with *N*_50_ of >0 and no contigs assigned to a genus outside *Bacillus* (*n *= 1,074) (E), and genomes with *N*_50_ of >100 kb and no contigs assigned to a genus outside *Bacillus* (i.e., the most stringent quality thresholds tested; *n *= 998) (F). Download FIG S2, PDF file, 0.1 MB. Copyright © 2020 Carroll et al. 2020 Carroll et al. This content is distributed under the terms of the Creative Commons Attribution 4.0 International license.

## DISCUSSION

When applied to bacteria, the concept of “species” is notoriously ambiguous, particularly in cases where it is intertwined with a phenotype, and even more so when that phenotype is an established component of the medical or industrial lexicon. Taxonomic definitions based on phenotype lack nuance in the omics era, as they ignore underpinning genomic diversity which can be leveraged to improve assessment of an isolate’s pathogenic potential or industrial utility. Furthermore, taxonomy based on phenotype can be ambiguous—and even misleading—when a trait is lost, gained, or not widespread throughout a lineage. A notable example is provided by botulinum neurotoxin (BoNT)-producing species, to which the Clostridium botulinum label has historically been applied, despite multiple genomospecies exhibiting BoNT production capabilities (6). Adherence to a nomenclature just for the sake of taxonomic rigor, however, can be equally problematic when a lineage has deep roots in medicine or industry. *Shigella* spp. and Escherichia coli, for example, constitute a single genomospecies but are considered to be distinct entities, despite genomic inconsistencies reflected in their nomenclature (7, 54, 55).

**FIG 6:**
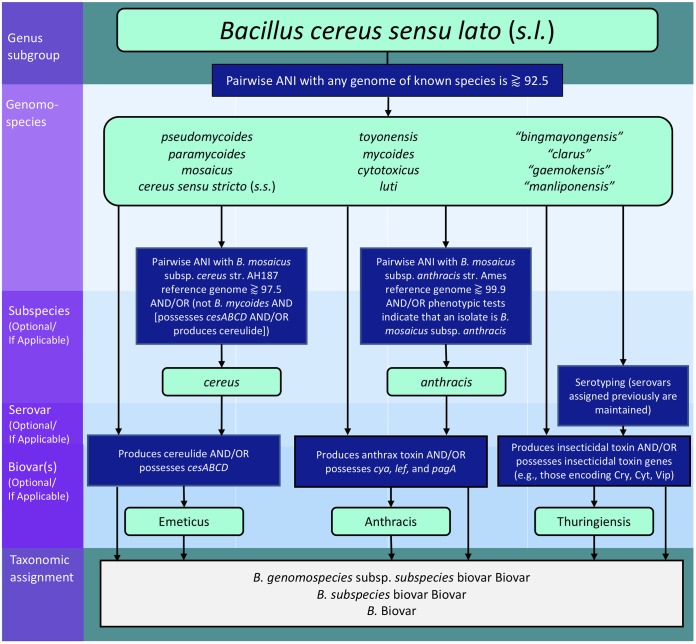
Taxonomic hierarchy for the proposed B. cereus group nomenclature. Taxonomic levels are listed in the left margin, with levels which are optional/not applicable to all organisms denoted as such. Rounded boxes shaded in light green correspond to possible taxonomic designations at their respective level, while blue boxes correspond to requirements that an isolate and/or its genome must meet to be assigned that designation. Possible forms which the final taxonomic assignment can take can be found in the gray box at the bottom of the chart.

An ideal taxonomy should be interpretable, without sacrificing the resolution provided by contemporary technologies. Several publications have appended the term “biovar” to species names to denote isolates which exhibit interesting phenotypes (e.g., anthrax-causing “B. cereus” as B. cereus biovar anthracis and Cry-producing *B. wiedmannii* as *B. wiedmannii* biovar thuringiensis) (41, 52). We therefore propose a taxonomic framework consisting of (i) an amended collection of genomospecies, corresponding to resolvable genomospecies obtained at ≈92.5 ANI; (ii) a formal collection of subspecies, which account for established lineages of medical importance; and (iii) a formalized and extended collection of biovars, which account for phenotypic heterogeneity (Fig. 6). Note that a recently proposed “genomovar” framework for B. cereus sensu stricto*/*B. thuringiensis (56) is not adopted here, due to the lack of genomospecies boundaries between their type strains (shown here and elsewhere [33–35], including the paper proposing the framework [56]), as well as the lack of a standardized species definition for B. thuringiensis (B. thuringiensis has been used to refer to any B. cereus group species capable of producing Bt toxins [29] or to the genomospecies formed by the B. thuringiensis type strain genome [56]).

### Proposed taxonomic nomenclature. (A) Genomospecies.

The B. cereus group currently consists of eight published genomospecies (designated I to VIII), four previously proposed genomospecies (designated ix to xii), and six putative novel genomospecies (designated xiii to xviii) (Fig. 5). A genome belongs to a genomospecies if it shares ⪆92.5 ANI with the genomospecies medoid genome (see Table S7 in the supplemental material). Due to the resolvability of genomospecies at this threshold, it follows that (i) a genome does not belong to a genomospecies if it shares ⪅92.5 ANI with the genomospecies medoid genome, (ii) two genomes belong to the same genomospecies if they share ⪆92.5 ANI with each other, and (iii) two genomes belong to different genomospecies if they share ⪅92.5 ANI with each other (i.e., in practice, a genomospecies medoid genome does not need to be used for genomospecies assignment, but rather any genome of known genomospecies; see Tables S6 and S7 for a comprehensive list of genomospecies assignments). When written, genomospecies names immediately follow the genus name (*Bacillus* or *B.*) and are italicized and lowercase.

### Published genomospecies.

**(I) *Bacillus pseudomycoides*.** The B. pseudomycoides genomospecies contained 111 genomes, including the genome of species type strain B. pseudomycoides strain DSM 12442. All genomes previously classified as B. pseudomycoides relative to the type strain at a 95 ANI threshold remain in this genomospecies, and no additional genomes belong to the genomospecies. As such, this genomospecies remains consistent with its previous classification, and its name remains unchanged.

**(II) *Bacillus paramycoides*.** The *B. paramycoides* genomospecies contained six genomes, including the genome of species type strain *B. paramycoides* strain NH24A2. All genomes previously classified as *B. paramycoides* relative to the type strain at a 95 ANI threshold remain in this genomospecies, and no additional genomes belong to the genomospecies. As such, this genomospecies remains consistent with its previous classification, and its name remains unchanged.

**(III) *Bacillus mosaicus*.** The *B. mosaicus* genomospecies contained 722 genomes, including type strains and reference genomes of species formerly known as *B. albus* (now *B. mosaicus* strain N35-10-2), B. anthracis (now *B. mosaicus* subsp. *anthracis* strain Ames; see “Subspecies” and “Biovars” below)*, B. mobilis* (now *B. mosaicus* strain 0711P9-1), *B. pacificus* (now *B. mosaicus* strain EB422), *B. paranthracis* (now *B. mosaicus* strain MN5), *B. tropicus* (now *B. mosaicus* strain N24), and *B. wiedmannii* (now *B. mosaicus* strain FSL W8-0169). Additionally, all members of the lineage formerly known as emetic “B. cereus” belong to *B. mosaicus* (see “Subspecies” and “Biovars” below). While the species formerly known as B. anthracis is the oldest described former species in this group, it is not proposed as the genomospecies name, as doing so could lead to incorrect assumptions of an isolate’s anthrax-causing potential. As such, the proposed genomospecies name (*mosaicus*) is chosen to reflect the diversity of lineages and phenotypes present among members of this genomospecies. All genomes previously assigned to the abovementioned former species using their respective type strain or reference genomes at a 95 ANI threshold belong to *B. mosaicus*.

**(IV) *Bacillus cereus sensu stricto*.** The B. cereus sensu stricto genomospecies contained 949 genomes, including those of type strains B. cereus sensu stricto (B. cereus sensu stricto strain ATCC 14579) and former species B. thuringiensis (now B. cereus sensu stricto serovar Berliner biovar Thuringiensis strain ATCC 10792; see “Biovars” below). B. cereus sensu stricto was chosen as the genomospecies name, with Thuringiensis proposed as a biovar to account for phenotypic heterogeneity within B. cereus sensu stricto, as well as the presence of insecticidal toxins in other genomospecies (see “Biovars” below). All genomes previously assigned to the species B. cereus sensu stricto and former species B. thuringiensis at a 95 ANI threshold using these type strains belong to B. cereus sensu stricto.

**(V) *Bacillus toyonensis*.** The *B. toyonensis* genomospecies contained 230 genomes, including the type strain of *B. toyonensis* (*B. toyonensis* strain BCT-7112). All genomes previously classified as *B. toyonensis* relative to the type strain at a 95 ANI threshold remain in this genomospecies, and no additional genomes belong to the genomospecies. As such, this genomospecies remains consistent with its previous classification, and its name remains unchanged.

**(VI) *Bacillus mycoides*.** The B. mycoides genomospecies contained 164 genomes, including the type strain of B. mycoides (B. mycoides strain DSM 2048), and former species *B. nitratireducens* (now B. mycoides strain 4049), *B. proteolyticus* (now B. mycoides strain TD42), and B. weihenstephanensis (now B. mycoides strain WSBC 10204). Additionally, all members of the lineages formerly known as emetic B. weihenstephanensis belong to B. mycoides (see “Biovars” below). B. mycoides was selected as the genomospecies name, as it is the oldest of the published former species described in this cluster (and remains consistent with taxonomic changes recently proposed by others [44]). All genomes previously assigned to the abovementioned species using their respective type strain or reference genomes and a 95 ANI threshold belong to B. mycoides.

**(VII) *Bacillus cytotoxicus*.** The *B. cytotoxicus* genomospecies contained 14 genomes, including the type strain of *B. cytotoxicus* (*B. cytotoxicus* strain NVH 391-98). All genomes previously classified as *B. cytotoxicus* relative to the type strain at a 95 ANI threshold remain in this genomospecies, and no additional genomes belong to the genomospecies. As such, this genomospecies remains consistent with its previous classification, and its name remains unchanged.

**(VIII) *Bacillus luti*.** The *B. luti* genomospecies contained nine genomes, including the type strain of *B. luti* (*B. luti* strain TD41). All genomes previously classified as *B. luti* relative to the type strain at a 95 ANI threshold remain in this genomospecies, and no additional genomes belong to the genomospecies. As such, this genomospecies remains consistent with its previous classification, and its name remains unchanged.

### Previously proposed putative genomospecies.

The following putative B. cereus group genomospecies which have been proposed previously remain unchanged: (IX) “*B. bingmayongensis*” (including type strain “*B. bingmayongensis*” strain FJAT-13831), (X) “*B. gaemokensis*” (including type strain “*B. gaemokensis*” strain KCTC 13318), (XI) “*B. manliponensis*” (including type strain “*B. manliponensis*” strain JCM 15802), and (XII) “*B. clarus*” (including type strain “*B. clarus*” strain ATCC 21929).

### Putative novel genomospecies.

Six putative genomospecies (xiii to xviii [Table S8]) have not been proposed as novel genomospecies. Future novel B. cereus group genomospecies should (i) share <92.5 ANI with all B. cereus group genomes and (ii) share ≥97% 16S rRNA gene similarity with known B. cereus group species (a definition used in previous studies [35]).

### (B) Subspecies.

The following subspecies are proposed to ensure that the medically important lineages formerly known as B. anthracis and emetic “B. cereus” remain interpretable. When written, subspecies names are italicized and lowercase and can optionally (i) be appended to the species name, after the nonitalicized delimiter “subspecies” or “subsp.,” prior to a serotype designation (if applicable); or (ii) follow the genus name (*Bacillus* or *B.*) directly, with the species name omitted, prior to a serotype designation (if applicable).

a. *Bacillus mosaicus* subsp. *anthracis* (can be written as *B. mosaicus* subsp. *anthracis*; B. anthracis) refers to the comparatively clonal lineage of former species B. anthracis commonly associated with anthrax illness. Isolates which are assigned to this subspecies (i) exhibit distinguishing phenotypic characteristics (e.g., lack of motility and lack of hemolysis on sheep red blood cell [RBC] agar) associated with the classical definition of B. anthracis as outlined in the *Bacteriological Analytical Manual* (BAM) chapter on B. cereus (2) and/or (ii) share ⪆99.9 ANI with former species reference genome B. anthracis strain Ames (now *B. mosaicus* subsp. *anthracis*; RefSeq accession no. GCF_000007845.1), a threshold previously identified for this lineage (5) which was replicated here. The use of the term “subspecies *anthracis*” does not indicate whether an isolate produces anthrax toxin or possesses the machinery required for anthrax toxin synthesis (see “biovar Anthracis” below).
b. *Bacillus mosaicus* subsp. *cereus* (can be written as *B. mosaicus* subsp. *cereus*; B. cereus) refers to the lineage formerly known as emetic “B. cereus.” All genomes possessing cereulide synthetase genes (*cesABCD*) which did not belong to the B. mycoides species cluster (see “Genomospecies” above) shared ≥97.5 ANI with the emetic reference strain formerly known as B. cereus strain AH187 (now *B. mosaicus* subsp. *cereus* biovar Emeticus; RefSeq accession no. GCF_000021225.1). As such, isolates assigned to this subspecies (i) produce cereulide and belong to the species *B. mosaicus*, (ii) possess *cesABCD* and belong to the species *B. mosaicus*, and/or (iii) share ⪆97.5 ANI with emetic reference genome B. cereus strain AH187 (now *B. mosaicus* subsp. *cereus* biovar Emeticus; RefSeq accession no. GCF_000021225.1). The use of the term “subspecies *cereus*” does not indicate whether an isolate produces cereulide or possesses the machinery required for cereulide synthesis (see “Biovar Emeticus” below).

### (C) Biovars.

The following biovars are proposed to account for phenotypes of clinical and industrial importance which can be distributed across species and heterogeneous in their appearance in individual lineages. While phenotypic evidence of a trait is ideal, biovars can be predicted at the genomic level. When written, (i) the first letter of the biovar is capitalized; (ii) the biovar name is not italicized; (iii) the biovar is appended to the end of a species, subspecies (if applicable), or serotype name (if applicable), following the nonitalicized delimiter “biovar”; (iv) if applicable, multiple biovars follow the nonitalicized, plural delimiter “biovars,” are listed in alphabetical order, and are each separated by a comma and a single space; (v) biovar(s) may follow the genus name (*Bacillus* or *B.*) directly, with the species, subspecies (if applicable), and serotype (if applicable) names omitted

a. Biovar Anthracis is applied to an isolate (i) known to produce anthrax toxin (preferred) and/or (ii) known to possess anthrax toxin-encoding genes *cya*, *lef*, and *pagA*. Capsular genes (e.g., *cap*, *has*, and *bps*) (21, 22, 57) are deliberately excluded from this definition as a conservative measure (i.e., to avoid cases in which an isolate might cause anthrax via a previously unknown capsule). Examples include *B. mosaicus* subsp. *anthracis* biovar Anthracis (i.e., anthrax-causing members of the “clonal” lineage often associated with anthrax disease; can be written as B. anthracis biovar Anthracis or *B.* Anthracis); *B. mosaicus* biovar Anthracis (i.e., anthrax-causing lineages formerly known as “anthrax-causing B. cereus”; can be written as *B.* Anthracis).
b. Biovar Emeticus is applied to an isolate known to produce cereulide (preferred) and/or to possess cereulide synthetase-encoding genes (*cesABCD*). Examples include *B. mosaicus* subsp. *cereus* biovar Emeticus (i.e., cereulide-producing lineages formerly known as emetic “B. cereus”; can be written as B. cereus biovar Emeticus or *B.* Emeticus) and B. mycoides biovar Emeticus (i.e., cereulide-producing lineages formerly known as “emetic B. weihenstephanensis”; can also be written as *B.* Emeticus).
c. Biovar Thuringiensis can be applied to an isolate known to produce one or more Bt toxins (e.g., Cry, Cyt, or Vip toxins; preferred) and/or to possess Bt toxin-encoding genes. Examples include *B. mosaicus* biovar Thuringiensis and B. cereus sensu stricto biovar Thuringiensis (both of which can be written as *B.* Thuringiensis).

The proposed taxonomy offers numerous advantages. Most importantly, it is consistent; it provides an explicit, standardized framework for taxonomic classification using genomic and/or phenotypic methods, and it resolves previous nomenclatural ambiguities. Second, the proposed taxonomy is backwards compatible with important medical and industrial taxonomic definitions. For example, any B. cereus group isolate capable of producing Bt toxins can be referred to as *B.* Thuringiensis, which is equivalent to the traditional species definition (29). All isolates capable of producing anthrax toxin can be referred to as *B.* Anthracis, while members of the “clonal” anthrax lineage remain B. anthracis (using subspecies notation). Finally, the proposed taxonomy is flexible and can be extended to account for additional lineages or phenotypes through the adoption of novel subspecies or biovars, respectively. For example, biovars can be proposed to describe B. cereus group members capable of causing diarrheal foodborne disease (i.e., biovar Cereus), as this disease involves multiple toxins and is not fully understood (58). The nomenclature proposed here not only provides a standardized framework for taxonomic classification which accounts for both phylogenomic diversity and phenotypic heterogeneity, but also serves as a model taxonomic framework which moves beyond arbitrary genomospecies thresholds while maintaining historical congruence.

## MATERIALS AND METHODS

### Acquisition and initial characterization of Bacillus cereus group genomes.

All genomes in the NCBI RefSeq Assembly database (59) which were submitted as one of 18 published B. cereus group species (35) were downloaded, along with the type strain genomes of three proposed effective B. cereus group species (60–62) (*n *= 2,231, accessed 19 November 2018) (see Tables S1 and S6 in the supplemental material). QUAST version 4.0 (63) was used to assess the quality of each genome, and BTyper version 2.3.2 (31) was used to detect B. cereus group virulence genes in each genome, using default minimum amino acid sequence identity and coverage thresholds (50% and 70%, respectively) (Table S6) (31, 64). Prokka version 1.12 (65) was used to annotate each genome, and the resulting coding sequences were used as input for the command-line implementation of BtToxin_scanner version 1.0 (BtToxin_scanner2.pl), which was used to identify Bt toxin genes in each genome using default settings (66).

### Calculation of pairwise ANI values, hierarchical clustering, and medoid genome identification.

FastANI version 1.0 (5) was used to calculate ANI values between each of 2,231 genomes (4,977,361 comparisons). To ensure that the breakpoints and shape of the distribution of pairwise ANI calculations were robust, genomes which (i) fell below various *N*_50_ thresholds (i.e., ≤10 kbp, 20 kbp, 50 kbp, and 100 kbp) and/or (ii) contained any contigs classified in domains other than *Bacteria*, phyla other than *Firmicutes*, and/or genera other than *Bacillus* using Kraken version 2.0.8-beta (67, 68) and the complete standard Kraken database (accessed 6 August 2019) were removed (Fig. S2). For medoid genome identification (described below), all genomes with *N*_50_ of >20 kbp in the original set of 2,231 RefSeq genomes were used in subsequent steps (*n *= 2,218) (Table S6 and Fig. S2).

The resulting pairwise ANI values were used to construct a similarity matrix, *S*_ANI_, using R version 3.6.0 (69) and the reshape2 package (70) as follows, where *n *= 2,218:

Let *g*_1_, *g*_2_, … *g_n_* be a set of *n* genomes, denoted by *G* (*G* = {*g*_1_, *g*_2_, … *g_n_*}). Similarity function *ANI*(*g_i_*, *g_j_*) denotes the ANI value shared by query and reference genomes *g_i_* and *g_j_*, respectively, where *ANI*: *G* × *G→*[0,100].

Similarity matrix *S*_ANI_ can be defined as *S*_ANI_ = (*s_ij_*); *s_ij_* = *ANI_ij_* = *ANI*(*g_i_*, *g_j_*).

Similarity matrix *S*_ANI_ was converted to a dissimilarity matrix, *D*_ANI_, as follows, where *J* denotes an *n *×* n* matrix where each element is equal to 1: *D*_ANI_ = 100*J* −*S*_ANI_.

*ANI* as a similarity function is not symmetric [i.e., for all *g_i_*, *g_j_*, *ANI*(*g_i_*, *g_j_*) ≠ *ANI*(*g_j_*, *g_i_*)], as minor differences between corresponding values in the upper and lower triangles of *D*_ANI_ existed: max[*d*(*g_i_*, *g_j_*), *d*(*g_j_*, *g_i_*)] = 0.504; min[*d*(*g_i_*, *g_j_*), *d*(*g_j_*, *g_i_*)] = 0; mean[*d*(*g_i_*, *g_j_*), *d*(*g_j_*, *g_i_*)] = 0.056; median[*d*(*g_i_*, *g_j_*), *d*(*g_j_*, *g_i_*)] = 0.046.

As such, *D*_ANI_ is not a symmetric matrix (i.e., *D*_ANI_ ≠ *D*_ANI_*^T^*). To coerce *D*_ANI_ to a symmetric matrix, $D_{\text{ANI}}^{\text{sym}}$, the following transformation was applied: $D_{\text{ANI}}^{\text{sym}}=0.5\left(D_{\text{ANI}}+\;D_{\text{ANI}}{}^{T}\right)$.

The hclust function in R’s stats package was used to perform average linkage hierarchical clustering, using $D_{\text{ANI}}^{\text{sym}}$ as the dissimilarity structure, and the resulting dendrogram was annotated using the ggplot2 (71), dendextend (72), and viridis (73) packages. Dendrogram clusters formed at various species thresholds (denoted here by *T_d_*, where *T_d_* = [5, 7.5], corresponding to ANI values of 95 and 92.5, respectively) were obtained by treating lineages which coalesced prior to *T_d_* as members of the same cluster (i.e., genomospecies) and those which did not as members of different clusters. Medoid genomes were identified within each cluster at each threshold, using the pam function in R’s cluster package (74) and $D_{\text{ANI}}^{\text{sym}}$ as a dissimilarity structure, where medoid genome is defined as

$$g_{\text{medoid}}={\text{arg min}}_{y\in \left\{g_{1},g_{2},\ldots g_{n}\right\}}\overset{n}{\underset{i=1}{\sum }}d\left(y,g_{i}\right)$$

where *d*(*g_i_*, *g_j_*) = 100 − *ANI*(*g_i_*, *g_j_*).

To construct a graph with each of 2,218 genomes represented as nodes and ANI values represented as weighted edges, $D_{\text{ANI}}^{\text{sym}}$ was converted to a symmetric similarity matrix, $S_{\text{ANI}}^{\text{sym}}$, as follows:

$$S_{\text{ANI}}^{\text{sym}}=-1(D_{\text{ANI}}^{\text{sym}}-100J).$$

The igraph (75) package in R was used to construct each graph, with $S_{\text{ANI}}^{\text{sym}}$ treated as an adjacency matrix, and edges with weights (i.e., ANI values) less than a similarity threshold *T_s_* (i.e., *T_s_* = [92.5, 95]) removed.

### Genomospecies assignment.

FastANI version 1.0 was used to assign each of 2,231 B. cereus group genomes to a genomospecies, using (i) species reference/type strain genomes (*n *= 21) (Table S1) and medoid genomes identified at (ii) 95 ANI (*n *= 36) (Table S3) and (iii) 92.5 ANI (*n *= 18) (Table S7) as reference genomes for each of three separate runs.

### Phylogeny construction.

Amino acid sequences of protein-encoding features produced by Prokka were used as input for OrthoFinder version 2.3.3 (76). Single-copy orthologous clusters (i.e., genes) present in all 2,231 genomes were identified using an iterative approach, in which OrthoFinder was used to identify single-copy genes core to *n* of the 2,231 genomes, sampled randomly without replacement, where *n *= 30 or *n *= 11 for 74 and 1 (the remainder) iteration(s), respectively. The union of single-copy genes present in all *n* genomes in each random sample of genomes was then queried again using OrthoFinder, which identified a total of 79 single-copy genes core to all 2,231 genomes. Nucleotide sequences of each of the 79 single-copy core genes were aligned using PRANK v.170427 (77). The resulting alignments were concatenated, and SNP-sites version 2.4.0 (78) was used to produce an alignment of variant sites, excluding gaps and ambiguous characters. IQ-TREE version 1.6.10 (79) was used to construct a maximum likelihood phylogeny, using the alignment of core single nucleotide polymorphisms (SNPs) detected in all 2,231 genomes. The GTR+G+ASC nucleotide substitution model (i.e., general time reversible model [80] with a gamma parameter [81] to allow rate heterogeneity among sites and an ascertainment bias correction [82] to account for the use of solely variant sites) was used, along with 1,000 replicates of the ultrafast bootstrap approximation (83). The resulting phylogeny was annotated in R using the ggplot2 (71), ape (84), phytools (85), phylobase (86), ggtree (87), and phangorn (88) packages.

### Data availability.

Accession numbers for all genomes queried in this study are available in Table S6. BTyper3, a command-line tool for characterizing B. cereus group genomes using the framework outlined here, is available at https://github.com/lmc297/BTyper3. An R package, bactaxR, is available for identifying medoid genomes and constructing plots using the methods described here at https://github.com/lmc297/bactaxR.

## References

[1] Tallent SM, Kotewicz KM, Strain EA, Bennett RW 2012 Efficient isolation and identification of *Bacillus cereus* group. J AOAC Int 95:446–451. doi:10.5740/jaoacint.11-251. 22649932

[2] Tallent SM, Rhodehamel EJ, Harmon SM, Bennett RW 2012 Bacillus cereus. Bacteriological analytical manual. US Food and Drug Administration, Washington, DC.

[3] Skerman VBD, McGowan V, Sneath PHA, Moore W 1989 Approved lists of bacterial names (amended). American Society for Microbiology, Washington, DC. 20806452

[4] Richter M, Rosselló-Móra R 2009 Shifting the genomic gold standard for the prokaryotic species definition. Proc Natl Acad Sci U S A 106:19126–19131. doi:10.1073/pnas.0906412106. 19855009 PMC2776425

[5] Jain C, Rodriguez RL, Phillippy AM, Konstantinidis KT, Aluru S 2018 High throughput ANI analysis of 90K prokaryotic genomes reveals clear species boundaries. Nat Commun 9:5114. doi:10.1038/s41467-018-07641-9. 30504855 PMC6269478

[6] Smith T, Williamson CHD, Hill K, Sahl J, Keim P 2018 Botulinum neurotoxin-producing bacteria. Isn’t it time that we called a species a species? mBio 9:e01469-18. doi:10.1128/mBio.01469-18. 30254123 PMC6156192

[7] Pettengill EA, Pettengill JB, Binet R 2015 Phylogenetic analyses of *Shigella* and enteroinvasive *Escherichia coli* for the identification of molecular epidemiological markers: whole-genome comparative analysis does not support distinct genera designation. Front Microbiol 6:1573. doi:10.3389/fmicb.2015.01573. 26834722 PMC4718091

[8] Forbes BA 2017 Mycobacterial taxonomy. J Clin Microbiol 55:380–383. doi:10.1128/JCM.01287-16. 27927928 PMC5277506

[9] Hoffmaster AR, Fitzgerald CC, Ribot E, Mayer LW, Popovic T 2002 Molecular subtyping of *Bacillus anthracis* and the 2001 bioterrorism-associated anthrax outbreak, United States. Emerg Infect Dis 8:1111–1116. doi:10.3201/eid0810.020394. 12396925 PMC2730295

[10] Takahashi H, Keim P, Kaufmann AF, Keys C, Smith KL, Taniguchi K, Inouye S, Kurata T 2004 *Bacillus anthracis* incident, Kameido, Tokyo, 1993. Emerg Infect Dis 10:117–120. doi:10.3201/eid1001.030238. 15112666 PMC3322761

[11] Abbara A, Brooks T, Taylor GP, Nolan M, Donaldson H, Manikon M, Holmes A 2014 Lessons for control of heroin-associated anthrax in Europe from 2009–2010 outbreak case studies, London, UK. Emerg Infect Dis 20:1115–1122. doi:10.3201/eid2007.131764. 24959910 PMC4073855

[12] Hanczaruk M, Reischl U, Holzmann T, Frangoulidis D, Wagner DM, Keim PS, Antwerpen MH, Meyer H, Grass G 2014 Injectional anthrax in heroin users, Europe, 2000–2012. Emerg Infect Dis 20:322–323. doi:10.3201/eid2002.120921. 24447525 PMC3901468

[13] Stenfors Arnesen LP, Fagerlund A, Granum PE 2008 From soil to gut: *Bacillus cereus* and its food poisoning toxins. FEMS Microbiol Rev 32:579–606. doi:10.1111/j.1574-6976.2008.00112.x. 18422617

[14] Bottone EJ 2010 *Bacillus cereus*, a volatile human pathogen. Clin Microbiol Rev 23:382–398. doi:10.1128/CMR.00073-09. 20375358 PMC2863360

[15] Jouzani GS, Valijanian E, Sharafi R 2017 *Bacillus thuringiensis*: a successful insecticide with new environmental features and tidings. Appl Microbiol Biotechnol 101:2691–2711. doi:10.1007/s00253-017-8175-y. 28235989

[16] Chattopadhyay P, Banerjee G, Mukherjee S 2017 Recent trends of modern bacterial insecticides for pest control practice in integrated crop management system. 3 Biotech 7:60. doi:10.1007/s13205-017-0717-6. PMC5428101 28444605

[17] Kamar R, Gohar M, Jehanno I, Rejasse A, Kallassy M, Lereclus D, Sanchis V, Ramarao N 2013 Pathogenic potential of *Bacillus cereus* strains as revealed by phenotypic analysis. J Clin Microbiol 51:320–323. doi:10.1128/JCM.02848-12. 23135929 PMC3536244

[18] Miller RA, Jian J, Beno SM, Wiedmann M, Kovac J 2018 Intraclade variability in toxin production and cytotoxicity of *Bacillus cereus* group type strains and dairy-associated isolates. Appl Environ Microbiol 84:e02479-17. doi:10.1128/AEM.02479-17. 29330180 PMC5835744

[19] Okinaka RT, Cloud K, Hampton O, Hoffmaster AR, Hill KK, Keim P, Koehler TM, Lamke G, Kumano S, Mahillon J, Manter D, Martinez Y, Ricke D, Svensson R, Jackson PJ 1999 Sequence and organization of pXO1, the large *Bacillus anthracis* plasmid harboring the anthrax toxin genes. J Bacteriol 181:6509–6515. doi:10.1128/JB.181.20.6509-6515.1999. 10515943 PMC103788

[20] Ezzell JW, Welkos SL 1999 The capsule of *Bacillus anthracis*, a review. J Appl Microbiol 87:250. doi:10.1046/j.1365-2672.1999.00881.x. 10475959

[21] Oh SY, Budzik JM, Garufi G, Schneewind O 2011 Two capsular polysaccharides enable *Bacillus cereus* G9241 to cause anthrax-like disease. Mol Microbiol 80:455–470. doi:10.1111/j.1365-2958.2011.07582.x. 21371137 PMC3538873

[22] Scarff JM, Seldina YI, Vergis JM, Ventura CL, O’Brien AD 2018 Expression and contribution to virulence of each polysaccharide capsule of *Bacillus cereus* strain G9241. PLoS One 13:e0202701. doi:10.1371/journal.pone.0202701. 30133532 PMC6105005

[23] Reyes-Ramírez A, Ibarra JE 2008 Plasmid patterns of *Bacillus thuringiensis* type strains. Appl Environ Microbiol 74:125–129. doi:10.1128/AEM.02133-07. 18024687 PMC2223206

[24] Meric G, Mageiros L, Pascoe B, Woodcock DJ, Mourkas E, Lamble S, Bowden R, Jolley KA, Raymond B, Sheppard SK 2018 Lineage-specific plasmid acquisition and the evolution of specialized pathogens in *Bacillus thuringiensis* and the *Bacillus cereus* group. Mol Ecol 27:1524–1540. doi:10.1111/mec.14546. 29509989 PMC5947300

[25] Gonzalez JM Jr, Brown BJ, Carlton BC 1982 Transfer of *Bacillus thuringiensis* plasmids coding for delta-endotoxin among strains of *B. thuringiensis* and *B. cereus*. Proc Natl Acad Sci U S A 79:6951–6955. doi:10.1073/pnas.79.22.6951. 6294667 PMC347252

[26] Ehling-Schulz M, Fricker M, Grallert H, Rieck P, Wagner M, Scherer S 2006 Cereulide synthetase gene cluster from emetic *Bacillus cereus*: structure and location on a mega virulence plasmid related to *Bacillus anthracis* toxin plasmid pXO1. BMC Microbiol 6:20. doi:10.1186/1471-2180-6-20. 16512902 PMC1459170

[27] Rasko DA, Rosovitz MJ, Okstad OA, Fouts DE, Jiang L, Cer RZ, Kolsto AB, Gill SR, Ravel J 2007 Complete sequence analysis of novel plasmids from emetic and periodontal *Bacillus cereus* isolates reveals a common evolutionary history among the *B. cereus*-group plasmids, including *Bacillus anthracis* pXO1. J Bacteriol 189:52–64. doi:10.1128/JB.01313-06. 17041058 PMC1797222

[28] Thorsen L, Hansen BM, Nielsen KF, Hendriksen NB, Phipps RK, Budde BB 2006 Characterization of emetic *Bacillus weihenstephanensis*, a new cereulide-producing bacterium. Appl Environ Microbiol 72:5118–5121. doi:10.1128/AEM.00170-06. 16820519 PMC1489381

[29] Zheng J, Gao Q, Liu L, Liu H, Wang Y, Peng D, Ruan L, Raymond B, Sun M 2017 Comparative genomics of *Bacillus thuringiensis* reveals a path to specialized exploitation of multiple invertebrate hosts. mBio 8:e00822-17. doi:10.1128/mBio.00822-17. 28790205 PMC5550751

[30] Klee SR, Brzuszkiewicz EB, Nattermann H, Bruggemann H, Dupke S, Wollherr A, Franz T, Pauli G, Appel B, Liebl W, Couacy-Hymann E, Boesch C, Meyer FD, Leendertz FH, Ellerbrok H, Gottschalk G, Grunow R, Liesegang H 2010 The genome of a *Bacillus* isolate causing anthrax in chimpanzees combines chromosomal properties of *B. cereus* with *B. anthracis* virulence plasmids. PLoS One 5:e10986. doi:10.1371/journal.pone.0010986. 20634886 PMC2901330

[31] Carroll LM, Kovac J, Miller RA, Wiedmann M 2017 Rapid, high-throughput identification of anthrax-causing and emetic *Bacillus cereus* group genome assemblies using BTyper, a computational tool for virulence-based classification of *Bacillus cereus* group isolates using nucleotide sequencing data. Appl Environ Microbiol 83:e01096-17. doi:10.1128/AEM.01096-17. 28625989 PMC5561296

[32] Guinebretiere MH, Auger S, Galleron N, Contzen M, De Sarrau B, De Buyser ML, Lamberet G, Fagerlund A, Granum PE, Lereclus D, De Vos P, Nguyen-The C, Sorokin A 2013 *Bacillus cytotoxicus* sp. nov. is a novel thermotolerant species of the *Bacillus cereus* group occasionally associated with food poisoning. Int J Syst Evol Microbiol 63:31–40. doi:10.1099/ijs.0.030627-0. 22328607

[33] Jiménez G, Urdiain M, Cifuentes A, López-López A, Blanch AR, Tamames J, Kämpfer P, Kolstø A-B, Ramón D, Martínez JF, Codoñer FM, Rosselló-Móra R 2013 Description of *Bacillus toyonensis* sp. nov., a novel species of the *Bacillus cereus* group, and pairwise genome comparisons of the species of the group by means of ANI calculations. Syst Appl Microbiol 36:383–391. doi:10.1016/j.syapm.2013.04.008. 23791203

[34] Miller RA, Beno SM, Kent DJ, Carroll LM, Martin NH, Boor KJ, Kovac J 2016 *Bacillus wiedmannii* sp. nov., a psychrotolerant and cytotoxic *Bacillus cereus* group species isolated from dairy foods and dairy environments. Int J Syst Evol Microbiol 66:4744–4753. doi:10.1099/ijsem.0.001421. 27520992 PMC5381181

[35] Liu Y, Du J, Lai Q, Zeng R, Ye D, Xu J, Shao Z 2017 Proposal of nine novel species of the *Bacillus cereus* group. Int J Syst Evol Microbiol 67:2499–2508. doi:10.1099/ijsem.0.001821. 28792367

[36] Ehling-Schulz M, Frenzel E, Gohar M 2015 Food-bacteria interplay: pathometabolism of emetic *Bacillus cereus*. Front Microbiol 6:704. doi:10.3389/fmicb.2015.00704. 26236290 PMC4500953

[37] Ehling-Schulz M, Svensson B, Guinebretiere MH, Lindback T, Andersson M, Schulz A, Fricker M, Christiansson A, Granum PE, Martlbauer E, Nguyen-The C, Salkinoja-Salonen M, Scherer S 2005 Emetic toxin formation of *Bacillus cereus* is restricted to a single evolutionary lineage of closely related strains. Microbiology 151:183–197. doi:10.1099/mic.0.27607-0. 15632437

[38] Ehling-Schulz M, Fricker M, Scherer S 2004 *Bacillus cereus*, the causative agent of an emetic type of food-borne illness. Mol Nutr Food Res 48:479–487. doi:10.1002/mnfr.200400055. 15538709

[39] Carroll LM, Wiedmann M, Mukherjee M, Nicholas DC, Mingle LA, Dumas NB, Cole JA, Kovac J 2019 Characterization of emetic and diarrheal *Bacillus cereus* strains from a 2016 foodborne outbreak using whole-genome sequencing: addressing the microbiological, epidemiological, and bioinformatic challenges. Front Microbiol 10:144. doi:10.3389/fmicb.2019.00144. 30809204 PMC6379260

[40] Marston CK, Ibrahim H, Lee P, Churchwell G, Gumke M, Stanek D, Gee JE, Boyer AE, Gallegos-Candela M, Barr JR, Li H, Boulay D, Cronin L, Quinn CP, Hoffmaster AR 2016 Anthrax toxin-expressing *Bacillus cereus* isolated from an anthrax-like eschar. PLoS One 11:e0156987. doi:10.1371/journal.pone.0156987. 27257909 PMC4892579

[41] Antonation KS, Grutzmacher K, Dupke S, Mabon P, Zimmermann F, Lankester F, Peller T, Feistner A, Todd A, Herbinger I, de Nys HM, Muyembe-Tamfun JJ, Karhemere S, Wittig RM, Couacy-Hymann E, Grunow R, Calvignac-Spencer S, Corbett CR, Klee SR, Leendertz FH 2016 *Bacillus cereus* biovar anthracis causing anthrax in sub-Saharan Africa—chromosomal monophyly and broad geographic distribution. PLoS Negl Trop Dis 10:e0004923. doi:10.1371/journal.pntd.0004923. 27607836 PMC5015827

[42] Wilson MK, Vergis JM, Alem F, Palmer JR, Keane-Myers AM, Brahmbhatt TN, Ventura CL, O’Brien AD 2011 *Bacillus cereus* G9241 makes anthrax toxin and capsule like highly virulent *B. anthracis* Ames but behaves like attenuated toxigenic nonencapsulated *B. anthracis* Sterne in rabbits and mice. Infect Immun 79:3012–3019. doi:10.1128/IAI.00205-11. 21576337 PMC3147598

[43] Mikesell P, Ivins BE, Ristroph JD, Dreier TM 1983 Evidence for plasmid-mediated toxin production in *Bacillus anthracis*. Infect Immun 39:371–376. doi:10.1128/IAI.39.1.371-376.1983. 6401695 PMC347948

[44] Liu Y, Lai Q, Shao Z 2018 Genome analysis-based reclassification of *Bacillus weihenstephanensis* as a later heterotypic synonym of *Bacillus mycoides*. Int J Syst Evol Microbiol 68:106–112. doi:10.1099/ijsem.0.002466. 29095136

[45] Dai Z, Sirard JC, Mock M, Koehler TM 1995 The *atxA* gene product activates transcription of the anthrax toxin genes and is essential for virulence. Mol Microbiol 16:1171–1181. doi:10.1111/j.1365-2958.1995.tb02340.x. 8577251

[46] Rodriguez RL, Gunturu S, Harvey WT, Rossello-Mora R, Tiedje JM, Cole JR, Konstantinidis KT 2018 The Microbial Genomes Atlas (MiGA) webserver: taxonomic and gene diversity analysis of Archaea and Bacteria at the whole genome level. Nucleic Acids Res 46:W282–W288. doi:10.1093/nar/gky467. 29905870 PMC6031002

[47] Vergnaud G, Girault G, Thierry S, Pourcel C, Madani N, Blouin Y 2016 Comparison of French and worldwide *Bacillus anthracis* strains favors a recent, post-Columbian origin of the predominant North-American clade. PLoS One 11:e0146216. doi:10.1371/journal.pone.0146216. 26901621 PMC4763433

[48] Sahl JW, Pearson T, Okinaka R, Schupp JM, Gillece JD, Heaton H, Birdsell D, Hepp C, Fofanov V, Noseda R, Fasanella A, Hoffmaster A, Wagner DM, Keim P 2016 A *Bacillus anthracis* genome sequence from the Sverdlovsk 1979 autopsy specimens. mBio 7:e01501-16. doi:10.1128/mBio.01501-16. 27677796 PMC5050339

[49] Agata N, Mori M, Ohta M, Suwan S, Ohtani I, Isobe M 1994 A novel dodecadepsipeptide, cereulide, isolated from *Bacillus cereus* causes vacuole formation in HEp-2 cells. FEMS Microbiol Lett 121:31–34. doi:10.1111/j.1574-6968.1994.tb07071.x. 8082824

[50] Hoton FM, Fornelos N, N’guessan E, Hu X, Swiecicka I, Dierick K, Jääskeläinen E, Salkinoja-Salonen M, Mahillon J 2009 Family portrait of *Bacillus cereus* and *Bacillus weihenstephanensis* cereulide-producing strains. Environ Microbiol Rep 1:177–183. doi:10.1111/j.1758-2229.2009.00028.x. 23765791

[51] Johler S, Kalbhenn EM, Heini N, Brodmann P, Gautsch S, Bağcioğlu M, Contzen M, Stephan R, Ehling-Schulz M 2018 Enterotoxin production of *Bacillus thuringiensis* isolates from biopesticides, foods, and outbreaks. Front Microbiol 9:1915. doi:10.3389/fmicb.2018.01915. 30190709 PMC6115515

[52] Lazarte JN, Lopez RP, Ghiringhelli PD, Beron CM 2018 *Bacillus wiedmannii* biovar thuringiensis: a specialized mosquitocidal pathogen with plasmids from diverse origins. Genome Biol Evol 10:2823–2833. doi:10.1093/gbe/evy211. 30285095 PMC6203079

[53] Acevedo MM, Carroll LM, Mukherjee M, Mills E, Xiaoli L, Dudley EG, Kovac J 2019 *Bacillus clarus* sp. nov. is a new *Bacillus cereus* group species isolated from soil bioRxiv 508077. doi:10.1101/508077. PMC7643830 33148822

[54] Chattaway MA, Schaefer U, Tewolde R, Dallman TJ, Jenkins C 2017 Identification of *Escherichia coli* and *Shigella* species from whole-genome sequences. J Clin Microbiol 55:616–623. doi:10.1128/JCM.01790-16. 27974538 PMC5277532

[55] Sahl JW, Morris CR, Emberger J, Fraser CM, Ochieng JB, Juma J, Fields B, Breiman RF, Gilmour M, Nataro JP, Rasko DA 2015 Defining the phylogenomics of *Shigella* species: a pathway to diagnostics. J Clin Microbiol 53:951–960. doi:10.1128/JCM.03527-14. 25588655 PMC4390639

[56] Baek I, Lee K, Goodfellow M, Chun J 2019 Comparative genomic and phylogenomic analyses clarify relationships within and between *Bacillus cereus* and *Bacillus thuringiensis*: Proposal for the recognition of two *Bacillus thuringiensis* genomovars. Front Microbiol 10:1978. doi:10.3389/fmicb.2019.01978. 31507580 PMC6716467

[57] Uchida I, Makino S, Sekizaki T, Terakado N 1997 Cross-talk to the genes for *Bacillus anthracis* capsule synthesis by *atxA*, the gene encoding the trans-activator of anthrax toxin synthesis. Mol Microbiol 23:1229–1240. doi:10.1046/j.1365-2958.1997.3041667.x. 9106214

[58] Doll VM, Ehling-Schulz M, Vogelmann R 2013 Concerted action of sphingomyelinase and non-hemolytic enterotoxin in pathogenic *Bacillus cereus*. PLoS One 8:e61404. doi:10.1371/journal.pone.0061404. 23613846 PMC3628865

[59] Pruitt KD, Tatusova T, Maglott DR 2007 NCBI reference sequences (RefSeq): a curated non-redundant sequence database of genomes, transcripts and proteins. Nucleic Acids Res 35:D61–D65. doi:10.1093/nar/gkl842. 17130148 PMC1716718

[60] Liu B, Liu G-H, Hu G-P, Sengonca C, Cetin S, Lin N-Q, Tang J-Y, Tang W-Q, Lin Y-Z 2014 *Bacillus bingmayongensis* sp. nov., isolated from the pit soil of Emperor Qin’s terra-cotta warriors in China. Antonie Van Leeuwenhoek 105:501–510. doi:10.1007/s10482-014-0150-3. 24370979

[61] Jung MY, Paek WK, Park I-S, Han J-R, Sin Y, Paek J, Rhee M-S, Kim H, Song HS, Chang Y-H 2010 *Bacillus gaemokensis* sp. nov., isolated from foreshore tidal flat sediment from the Yellow Sea. J Microbiol 48:867–871. doi:10.1007/s12275-010-0148-0. 21221948

[62] Jung MY, Kim JS, Paek WK, Lim J, Lee H, Kim PI, Ma JY, Kim W, Chang YH 2011 *Bacillus manliponensis* sp. nov., a new member of the *Bacillus cereus* group isolated from foreshore tidal flat sediment. J Microbiol 49:1027–1032. doi:10.1007/s12275-011-1049-6. 22203569

[63] Gurevich A, Saveliev V, Vyahhi N, Tesler G 2013 QUAST: quality assessment tool for genome assemblies. Bioinformatics 29:1072–1075. doi:10.1093/bioinformatics/btt086. 23422339 PMC3624806

[64] Kovac J, Miller RA, Carroll LM, Kent DJ, Jian J, Beno SM, Wiedmann M 2016 Production of hemolysin BL by *Bacillus cereus* group isolates of dairy origin is associated with whole-genome phylogenetic clade. BMC Genomics 17:581. doi:10.1186/s12864-016-2883-z. 27507015 PMC4979109

[65] Seemann T 2014 Prokka: rapid prokaryotic genome annotation. Bioinformatics 30:2068–2069. doi:10.1093/bioinformatics/btu153. 24642063

[66] Ye W, Zhu L, Liu Y, Crickmore N, Peng D, Ruan L, Sun M 2012 Mining new crystal protein genes from *Bacillus thuringiensis* on the basis of mixed plasmid-enriched genome sequencing and a computational pipeline. Appl Environ Microbiol 78:4795–4801. doi:10.1128/AEM.00340-12. 22544259 PMC3416374

[67] Wood DE, Salzberg SL 2014 Kraken: ultrafast metagenomic sequence classification using exact alignments. Genome Biol 15:R46. doi:10.1186/gb-2014-15-3-r46. 24580807 PMC4053813

[68] Wood DE, Lu J, Langmead B 2019 Improved metagenomic analysis with Kraken 2. bioRxiv 762302. doi:10.1101/762302. PMC6883579 31779668

[69] R Core Team. 2018 R: a language and environment for statistical computing. R Foundation for Statistical Computing, Vienna, Austria https://www.R-project.org/.

[70] Wickham H 2007 Reshaping data with the reshape package. J Stat Softw 21:1–20.

[71] Wickham H 2009 Ggplot2: elegant graphics for data analysis. Springer, New York, NY.

[72] Galili T 2015 dendextend: an R package for visualizing, adjusting and comparing trees of hierarchical clustering. Bioinformatics 31:3718–3720. doi:10.1093/bioinformatics/btv428. 26209431 PMC4817050

[73] Garnier S 2018 viridis: default color maps from ‘matplotlib’, vR package version 0.5.1. https://CRAN.R-project.org/package=viridis.

[74] Maechler M, Rousseeuw P, Struyf A, Hubert M, Hornik K 2017 cluster: cluster analysis basics and extensions, v2.0.6.

[75] Csardi G, Nepusz T 2006 The igraph software package for complex network research. InterJournal Complex Systems:1695.

[76] Emms DM, Kelly S 2015 OrthoFinder: solving fundamental biases in whole genome comparisons dramatically improves orthogroup inference accuracy. Genome Biol 16:157. doi:10.1186/s13059-015-0721-2. 26243257 PMC4531804

[77] Loytynoja A 2014 Phylogeny-aware alignment with PRANK. Methods Mol Biol 1079:155–170. doi:10.1007/978-1-62703-646-7_10. 24170401

[78] Page AJ, Taylor B, Delaney AJ, Soares J, Seemann T, Keane JA, Harris SR 2016 SNP-sites: rapid efficient extraction of SNPs from multi-FASTA alignments. Microb Genom 2:e000056. doi:10.1099/mgen.0.000056. 28348851 PMC5320690

[79] Nguyen LT, Schmidt HA, von Haeseler A, Minh BQ 2015 IQ-TREE: a fast and effective stochastic algorithm for estimating maximum-likelihood phylogenies. Mol Biol Evol 32:268–274. doi:10.1093/molbev/msu300. 25371430 PMC4271533

[80] Tavare S 1986 Some probabilistic and statistical problems in the analysis of DNA sequences. Lect Math Life Sci 17:57–86.

[81] Yang Z 1994 Maximum likelihood phylogenetic estimation from DNA sequences with variable rates over sites: approximate methods. J Mol Evol 39:306–314. doi:10.1007/bf00160154. 7932792

[82] Lewis PO 2001 A likelihood approach to estimating phylogeny from discrete morphological character data. Syst Biol 50:913–925. doi:10.1080/106351501753462876. 12116640

[83] Hoang DT, Chernomor O, von Haeseler A, Minh BQ, Vinh LS 2018 UFBoot2: improving the ultrafast bootstrap approximation. Mol Biol Evol 35:518–522. doi:10.1093/molbev/msx281. 29077904 PMC5850222

[84] Paradis E, Schliep K 2019 ape 5.0: an environment for modern phylogenetics and evolutionary analyses in R. Bioinformatics 35:526–528. doi:10.1093/bioinformatics/bty633. 30016406

[85] Revell LJ 2012 phytools: an R package for phylogenetic comparative biology (and other things). Methods Ecol Evol 3:217–223. doi:10.1111/j.2041-210X.2011.00169.x.

[86] R Hackathon. 2017 phylobase: base package for phylogenetic structures and comparative data, v0.8.4. https://CRAN.R-project.org/package=phylobase.

[87] Yu G, Smith DK, Zhu H, Guan Y, Lam T 2017 ggtree: an R package for visualization and annotation of phylogenetic trees with their covariates and other associated data. Methods Ecol Evol 8:28–36. doi:10.1111/2041-210X.12628.

[88] Schliep KP 2011 phangorn: phylogenetic analysis in R. Bioinformatics 27:592–593. doi:10.1093/bioinformatics/btq706. 21169378 PMC3035803

