# Supplementary material for: Proposal of a Taxonomic Nomenclature for the Bacillus cereus Group Which Reconciles Genomic Definitions of Bacterial Species with Clinical and Industrial Phenotypes
Source: mBio. 2020 Feb 25;11(1):e00034-20. doi: 10.1128/mBio.00034-20 (PMC7042689; doi:10.1128/mBio.00034-20)
Supplement: FIG S1 [file mBio.00034-20-sf001.pdf]

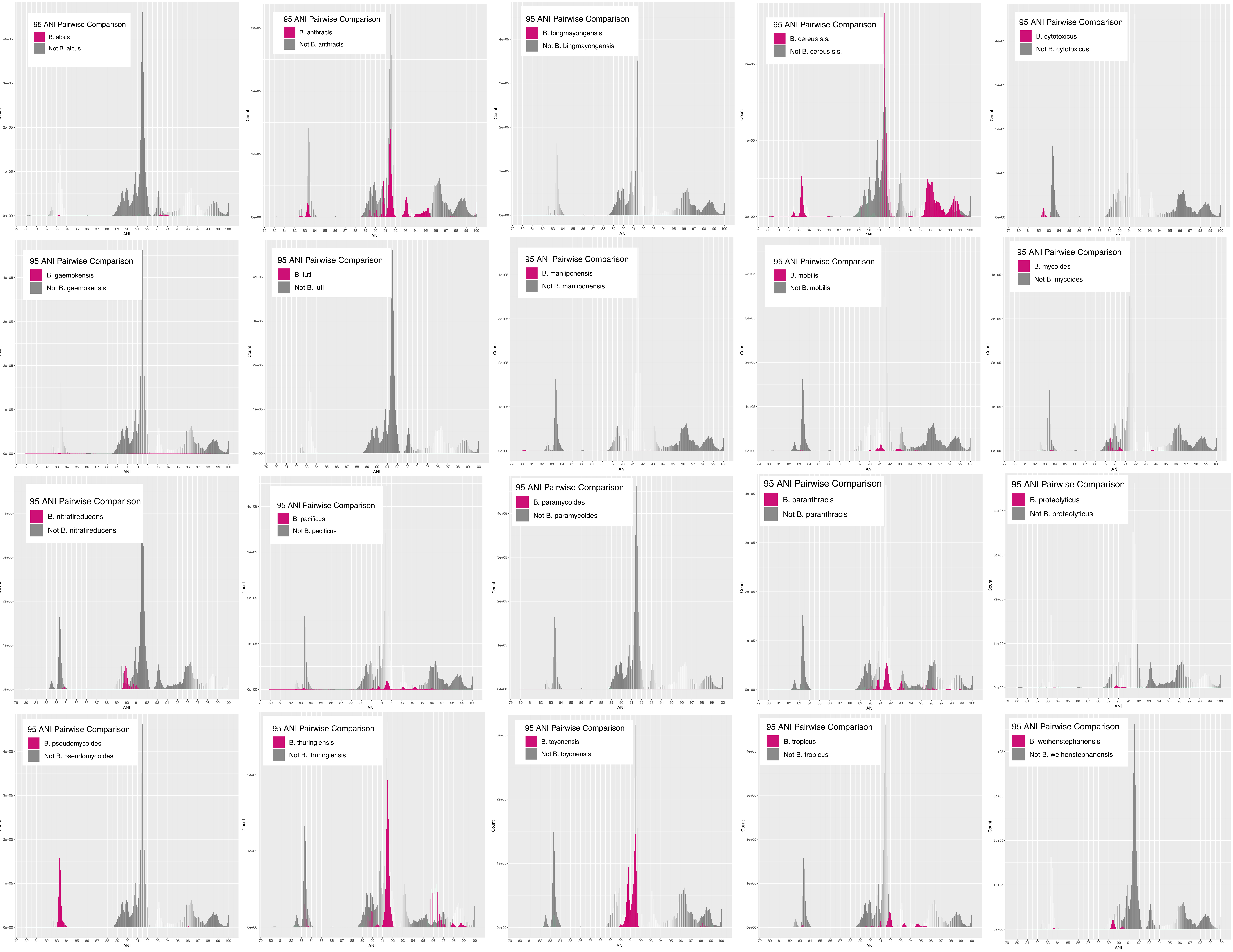

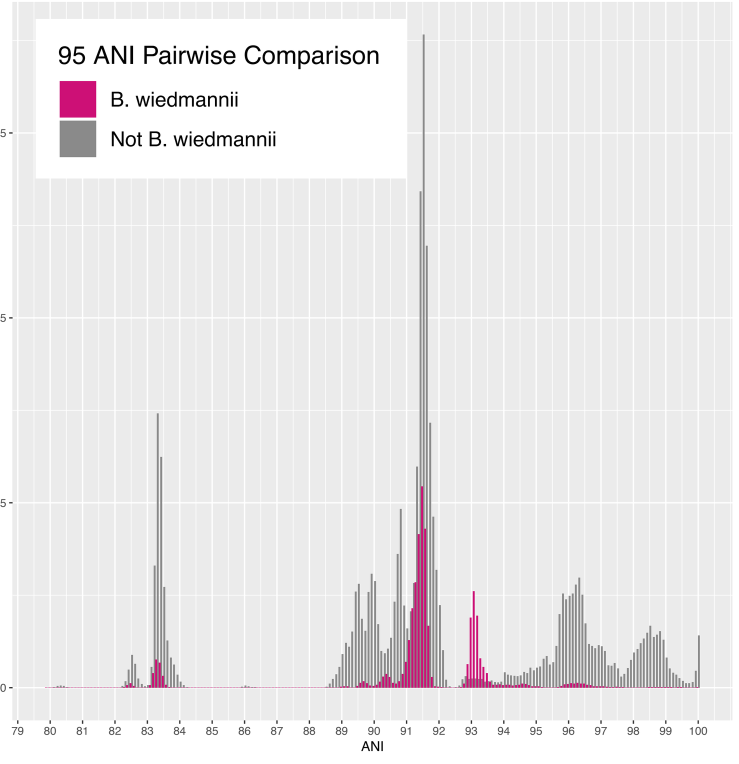

Supplemental Figure S1. Histograms of pairwise ANI values between 2,218 *B. cereus* group genomes with N50 > 20 Kbp. For each of 18 published species and 3 proposed putative species, ANI values calculated between two genomes for which one or more genome(s) shared ≥ 95 ANI with the species type strain or reference genome are colored in pink. ANI values calculated between two genomes for which both genomes shared < 95 ANI with the species type strain/reference genome are colored in gray. See Supplemental Table S1 for details regarding species type strain genomes.
